# Supplementary figures and images for: Hemagglutination Inhibition (HAI) antibody landscapes after vaccination with H7Nx virus like particles
Source: PLoS One. 2021 Mar 18;16(3):e0246613. doi: 10.1371/journal.pone.0246613 (PMC7971484; doi:10.1371/journal.pone.0246613)

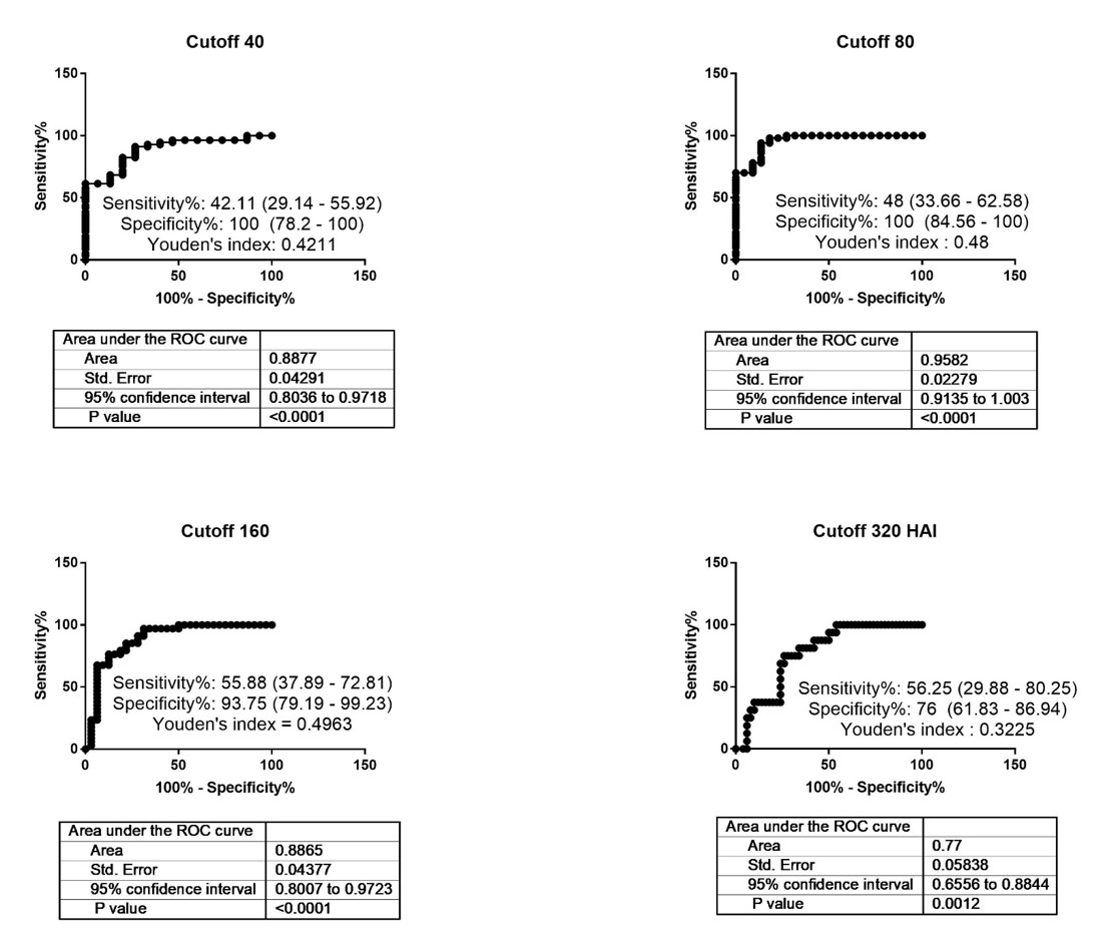

Supplement: S1 Fig — The plots of sensitivity% versus false positive rate (100-specificity%) of each cut-off were connected to form the ROC curve. Sensitivity = number of mice which showed hemagglutination inhibition (HAI) titer ≥ cut-off and was protected from the challenge study/all protected mice, Specificity = number of mice which showed hemagglutination inhibition (HAI) titer < cut-off and unprotected from the challenge study/ number of all unprotected mice, Youden’s index = Sensitivity + Specificity -1. (TIF) [file pone.0246613.s001.tif]

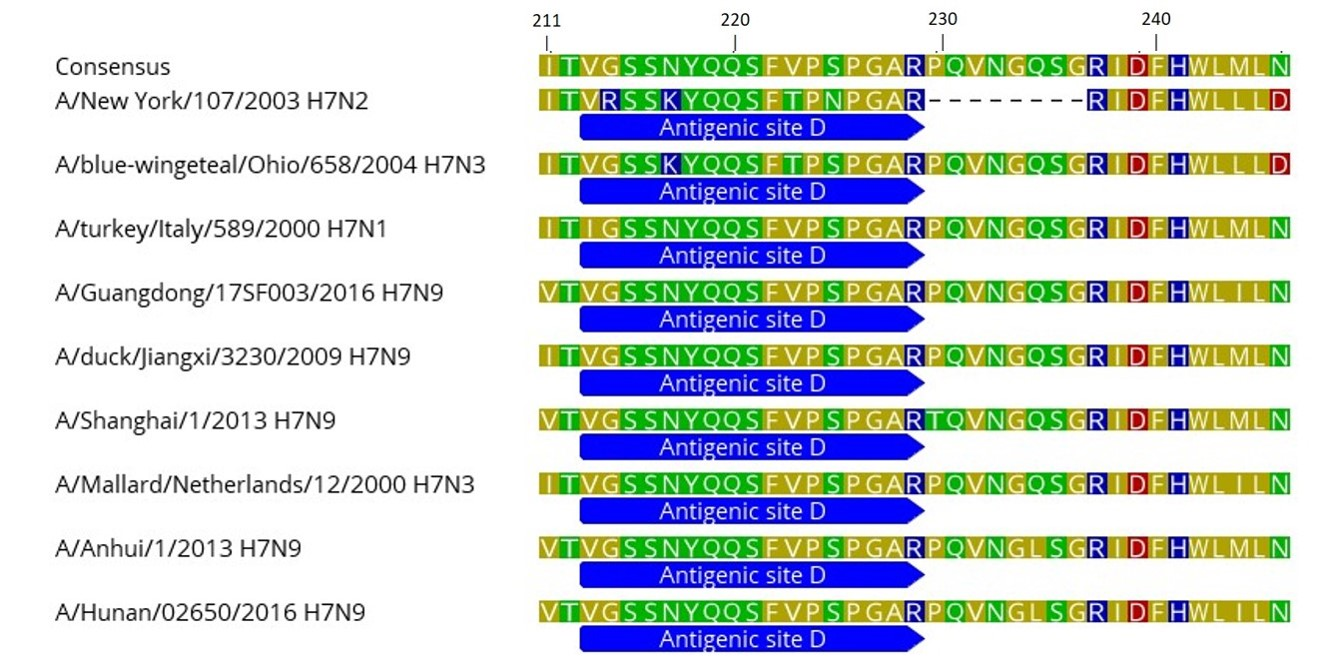

Supplement: S2 Fig — (TIF) [file pone.0246613.s002.tif]

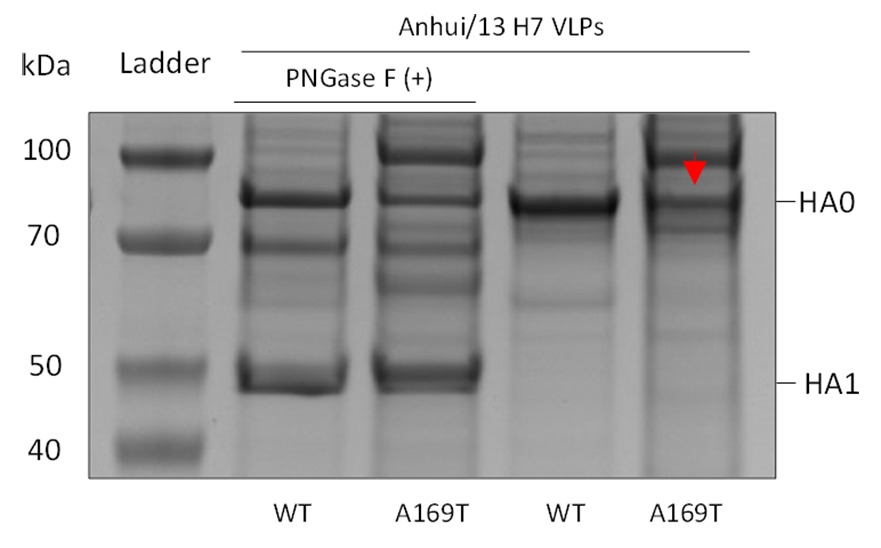

Supplement: S3 Fig — The wild type (WT)-, and A169T mutant Anhui/13 VLPs were characterized by comassie blue stained SDS-PAGE. VLPs loaded on left two lanes were pre-treated with PNGaseF to remove the N-linked glycans. Red arrow indicates HA0 band for WT Anhui/13 VLPs. (TIF) [file pone.0246613.s003.tif]
