# Supplementary material for: Hemagglutination Inhibition (HAI) antibody landscapes after vaccination with H7Nx virus like particles
Source: PLoS One. 2021 Mar 18;16(3):e0246613. doi: 10.1371/journal.pone.0246613 (PMC7971484; doi:10.1371/journal.pone.0246613)
Supplement: S1 Table — (DOCX) [file pone.0246613.s004.docx]

**S1 Table. Sensitivity and specificity of 1:40 HAI cut off to predict protection defined by 90-100% of original body weight**

| Protection if  % body weight | Sensitivity (%) | | Specificity (%) | |
| --- | --- | --- | --- | --- |
|  | Average | 95% CI | Average | 95% CI |
| > 90.04 | 64.91 | 51.13% to 77.09% | 86.67 | 59.54% to 98.34% |
| > 90.29 | 63.16 | 49.34% to 75.55% | 86.67 | 59.54% to 98.34% |
| > 90.51 | 61.4 | 47.57% to 74.00% | 86.67 | 59.54% to 98.34% |
| > 90.56 | 61.4 | 47.57% to 74.00% | 93.33 | 68.05% to 99.83% |
| > 90.83 | 61.4 | 47.57% to 74.00% | 100 | 78.20% to 100.0% |
| > 91.66 | 57.89 | 44.08% to 70.86% | 100 | 78.20% to 100.0% |
| > 92.38 | 56.14 | 42.36% to 69.26% | 100 | 78.20% to 100.0% |
| > 92.64 | 54.39 | 40.66% to 67.64% | 100 | 78.20% to 100.0% |
| > 92.85 | 52.63 | 38.97% to 66.02% | 100 | 78.20% to 100.0% |
| > 93.05 | 50.88 | 37.29% to 64.37% | 100 | 78.20% to 100.0% |
| > 93.77 | 49.12 | 35.63% to 62.71% | 100 | 78.20% to 100.0% |
| > 94.40 | 47.37 | 33.98% to 61.03% | 100 | 78.20% to 100.0% |
| > 94.72 | 43.86 | 30.74% to 57.64% | 100 | 78.20% to 100.0% |
| > 95.11 | 42.11 | 29.14% to 55.92% | 100 | 78.20% to 100.0% |
| > 95.82 | 38.6 | 26.00% to 52.43% | 100 | 78.20% to 100.0% |
| > 96.60 | 36.84 | 24.45% to 50.66% | 100 | 78.20% to 100.0% |
| > 96.92 | 35.09 | 22.91% to 48.87% | 100 | 78.20% to 100.0% |
| > 97.20 | 33.33 | 21.40% to 47.06% | 100 | 78.20% to 100.0% |
| > 97.41 | 31.58 | 19.91% to 45.24% | 100 | 78.20% to 100.0% |
| > 97.68 | 29.82 | 18.43% to 43.40% | 100 | 78.20% to 100.0% |
| > 98.11 | 28.07 | 16.97% to 41.54% | 100 | 78.20% to 100.0% |
| > 98.43 | 26.32 | 15.54% to 39.66% | 100 | 78.20% to 100.0% |
| > 99.08 | 24.56 | 14.13% to 37.76% | 100 | 78.20% to 100.0% |
| > 99.87 | 22.81 | 12.74% to 35.84% | 100 | 78.20% to 100.0% |
| > 100.3 | 19.3 | 10.05% to 31.91% | 100 | 78.20% to 100.0% |
| > 100.5 | 17.54 | 8.747% to 29.91% | 100 | 78.20% to 100.0% |
| > 100.7 | 15.79 | 7.483% to 27.87% | 100 | 78.20% to 100.0% |
| > 100.7 | 14.04 | 6.259% to 25.79% | 100 | 78.20% to 100.0% |
| > 100.8 | 12.28 | 5.083% to 23.68% | 100 | 78.20% to 100.0% |
| > 101.3 | 10.53 | 3.962% to 21.52% | 100 | 78.20% to 100.0% |
| > 102.1 | 8.772 | 2.910% to 19.30% | 100 | 78.20% to 100.0% |
| > 102.4 | 5.263 | 1.099% to 14.62% | 100 | 78.20% to 100.0% |
| > 102.9 | 3.509 | 0.4278% to 12.11% | 100 | 78.20% to 100.0% |
