# Supplementary material for: Hemagglutination Inhibition (HAI) antibody landscapes after vaccination with H7Nx virus like particles
Source: PLoS One. 2021 Mar 18;16(3):e0246613. doi: 10.1371/journal.pone.0246613 (PMC7971484; doi:10.1371/journal.pone.0246613)
Supplement: S2 Table — (DOCX) [file pone.0246613.s005.docx]

**S2 Table. Sensitivity and specificity of 1:80 HAI cut off to predict protection defined by 90-100% of original body weight**

| Protection if  % body weight | Sensitivity (%) | | Specificity (%) | |
| --- | --- | --- | --- | --- |
|  | Average | 95% CI | Average | 95% CI |
| > 90.04 | 74 | 59.66% to 85.37% | 90.91 | 70.84% to 98.88% |
| > 90.29 | 72 | 57.51% to 83.77% | 90.91 | 70.84% to 98.88% |
| > 90.51 | 70 | 55.39% to 82.14% | 90.91 | 70.84% to 98.88% |
| > 90.56 | 70 | 55.39% to 82.14% | 95.45 | 77.16% to 99.88% |
| > 90.83 | 70 | 55.39% to 82.14% | 100 | 84.56% to 100.0% |
| > 91.66 | 66 | 51.23% to 78.79% | 100 | 84.56% to 100.0% |
| > 92.38 | 64 | 49.19% to 77.08% | 100 | 84.56% to 100.0% |
| > 92.64 | 62 | 47.17% to 75.35% | 100 | 84.56% to 100.0% |
| > 92.85 | 60 | 45.18% to 73.59% | 100 | 84.56% to 100.0% |
| > 93.05 | 58 | 43.21% to 71.81% | 100 | 84.56% to 100.0% |
| > 93.77 | 56 | 41.25% to 70.01% | 100 | 84.56% to 100.0% |
| > 94.40 | 54 | 39.32% to 68.19% | 100 | 84.56% to 100.0% |
| > 94.72 | 50 | 35.53% to 64.47% | 100 | 84.56% to 100.0% |
| > 95.11 | 48 | 33.66% to 62.58% | 100 | 84.56% to 100.0% |
| > 95.82 | 44 | 29.99% to 58.75% | 100 | 84.56% to 100.0% |
| > 96.60 | 42 | 28.19% to 56.79% | 100 | 84.56% to 100.0% |
| > 96.92 | 40 | 26.41% to 54.82% | 100 | 84.56% to 100.0% |
| > 97.20 | 38 | 24.65% to 52.83% | 100 | 84.56% to 100.0% |
| > 97.41 | 36 | 22.92% to 50.81% | 100 | 84.56% to 100.0% |
| > 97.68 | 34 | 21.21% to 48.77% | 100 | 84.56% to 100.0% |
| > 98.11 | 32 | 19.52% to 46.70% | 100 | 84.56% to 100.0% |
| > 98.43 | 30 | 17.86% to 44.61% | 100 | 84.56% to 100.0% |
| > 99.08 | 28 | 16.23% to 42.49% | 100 | 84.56% to 100.0% |
| > 99.87 | 26 | 14.63% to 40.34% | 100 | 84.56% to 100.0% |
| > 100.3 | 22 | 11.53% to 35.96% | 100 | 84.56% to 100.0% |
| > 100.5 | 20 | 10.03% to 33.72% | 100 | 84.56% to 100.0% |
| > 100.7 | 18 | 8.576% to 31.44% | 100 | 84.56% to 100.0% |
| > 100.7 | 16 | 7.170% to 29.11% | 100 | 84.56% to 100.0% |
| > 100.8 | 14 | 5.819% to 26.74% | 100 | 84.56% to 100.0% |
| > 101.3 | 12 | 4.534% to 24.31% | 100 | 84.56% to 100.0% |
| > 102.1 | 10 | 3.328% to 21.81% | 100 | 84.56% to 100.0% |
| > 102.4 | 6 | 1.255% to 16.55% | 100 | 84.56% to 100.0% |
| > 102.9 | 4 | 0.4881% to 13.71% | 100 | 84.56% to 100.0% |
