# Supplementary material for: Hemagglutination Inhibition (HAI) antibody landscapes after vaccination with H7Nx virus like particles
Source: PLoS One. 2021 Mar 18;16(3):e0246613. doi: 10.1371/journal.pone.0246613 (PMC7971484; doi:10.1371/journal.pone.0246613)
Supplement: S3 Table — (DOCX) [file pone.0246613.s006.docx]

**S3 Table. Sensitivity and specificity of 1:160 HAI cut off to predict protection defined by 90-100% of original body weight**

| Protection if  % body weight | Sensitivity (%) | | Specificity (%) | |
| --- | --- | --- | --- | --- |
|  | Average | 95% CI | Average | 95% CI |
| > 90.04 | 79.41 | 62.10% to 91.30% | 78.13 | 60.03% to 90.72% |
| > 90.29 | 79.41 | 62.10% to 91.30% | 81.25 | 63.56% to 92.79% |
| > 90.51 | 76.47 | 58.83% to 89.25% | 81.25 | 63.56% to 92.79% |
| > 90.56 | 76.47 | 58.83% to 89.25% | 84.38 | 67.21% to 94.72% |
| > 90.83 | 76.47 | 58.83% to 89.25% | 87.5 | 71.01% to 96.49% |
| > 91.66 | 73.53 | 55.64% to 87.12% | 87.5 | 71.01% to 96.49% |
| > 92.38 | 70.59 | 52.52% to 84.90% | 87.5 | 71.01% to 96.49% |
| > 92.64 | 67.65 | 49.47% to 82.61% | 87.5 | 71.01% to 96.49% |
| > 92.85 | 67.65 | 49.47% to 82.61% | 90.63 | 74.98% to 98.02% |
| > 93.05 | 67.65 | 49.47% to 82.61% | 93.75 | 79.19% to 99.23% |
| > 93.77 | 64.71 | 46.49% to 80.25% | 93.75 | 79.19% to 99.23% |
| > 94.40 | 61.76 | 43.56% to 77.83% | 93.75 | 79.19% to 99.23% |
| > 94.72 | 58.82 | 40.70% to 75.35% | 93.75 | 79.19% to 99.23% |
| > 95.11 | 55.88 | 37.89% to 72.81% | 93.75 | 79.19% to 99.23% |
| > 95.82 | 52.94 | 35.13% to 70.22% | 93.75 | 79.19% to 99.23% |
| > 96.60 | 50 | 32.43% to 67.57% | 93.75 | 79.19% to 99.23% |
| > 96.92 | 47.06 | 29.78% to 64.87% | 93.75 | 79.19% to 99.23% |
| > 97.20 | 44.12 | 27.19% to 62.11% | 93.75 | 79.19% to 99.23% |
| > 97.41 | 41.18 | 24.65% to 59.30% | 93.75 | 79.19% to 99.23% |
| > 97.68 | 38.24 | 22.17% to 56.44% | 93.75 | 79.19% to 99.23% |
| > 98.11 | 35.29 | 19.75% to 53.51% | 93.75 | 79.19% to 99.23% |
| > 98.43 | 32.35 | 17.39% to 50.53% | 93.75 | 79.19% to 99.23% |
| > 99.08 | 29.41 | 15.10% to 47.48% | 93.75 | 79.19% to 99.23% |
| > 99.87 | 26.47 | 12.88% to 44.36% | 93.75 | 79.19% to 99.23% |
| > 100.3 | 23.53 | 10.75% to 41.17% | 93.75 | 79.19% to 99.23% |
| > 100.5 | 23.53 | 10.75% to 41.17% | 96.88 | 83.78% to 99.92% |
| > 100.7 | 20.59 | 8.702% to 37.90% | 96.88 | 83.78% to 99.92% |
| > 100.7 | 17.65 | 6.764% to 34.53% | 96.88 | 83.78% to 99.92% |
| > 100.8 | 14.71 | 4.953% to 31.06% | 96.88 | 83.78% to 99.92% |
| > 101.3 | 11.76 | 3.300% to 27.45% | 96.88 | 83.78% to 99.92% |
| > 102.1 | 8.824 | 1.858% to 23.68% | 96.88 | 83.78% to 99.92% |
| > 102.4 | 5.882 | 0.7205% to 19.68% | 96.88 | 83.78% to 99.92% |
| > 102.9 | 2.941 | 0.07444% to 15.33% | 96.88 | 83.78% to 99.92% |
