# Supplementary material for: Hemagglutination Inhibition (HAI) antibody landscapes after vaccination with H7Nx virus like particles
Source: PLoS One. 2021 Mar 18;16(3):e0246613. doi: 10.1371/journal.pone.0246613 (PMC7971484; doi:10.1371/journal.pone.0246613)
Supplement: S4 Table — (DOCX) [file pone.0246613.s007.docx]

**S4 Table. Sensitivity and specificity of 1:320 HAI cut off to predict protection defined by 90-100% of original body weight**

| Protection if  % body weight | Sensitivity (%) | | Specificity (%) | |
| --- | --- | --- | --- | --- |
|  | Average | 95% CI | Average | 95% CI |
| > 90.04 | 81.25 | 54.35% to 95.95% | 58 | 43.21% to 71.81% |
| > 90.29 | 81.25 | 54.35% to 95.95% | 60 | 45.18% to 73.59% |
| > 90.51 | 81.25 | 54.35% to 95.95% | 62 | 47.17% to 75.35% |
| > 90.56 | 81.25 | 54.35% to 95.95% | 64 | 49.19% to 77.08% |
| > 90.83 | 81.25 | 54.35% to 95.95% | 66 | 51.23% to 78.79% |
| > 91.66 | 75 | 47.62% to 92.73% | 66 | 51.23% to 78.79% |
| > 92.38 | 75 | 47.62% to 92.73% | 68 | 53.30% to 80.48% |
| > 92.64 | 75 | 47.62% to 92.73% | 70 | 55.39% to 82.14% |
| > 92.85 | 75 | 47.62% to 92.73% | 72 | 57.51% to 83.77% |
| > 93.05 | 75 | 47.62% to 92.73% | 74 | 59.66% to 85.37% |
| > 93.77 | 68.75 | 41.34% to 88.98% | 74 | 59.66% to 85.37% |
| > 94.40 | 68.75 | 41.34% to 88.98% | 76 | 61.83% to 86.94% |
| > 94.72 | 62.5 | 35.43% to 84.80% | 76 | 61.83% to 86.94% |
| > 95.11 | 56.25 | 29.88% to 80.25% | 76 | 61.83% to 86.94% |
| > 95.82 | 50 | 24.65% to 75.35% | 76 | 61.83% to 86.94% |
| > 96.60 | 43.75 | 19.75% to 70.12% | 76 | 61.83% to 86.94% |
| > 96.92 | 37.5 | 15.20% to 64.57% | 76 | 61.83% to 86.94% |
| > 97.20 | 37.5 | 15.20% to 64.57% | 78 | 64.04% to 88.47% |
| > 97.41 | 37.5 | 15.20% to 64.57% | 80 | 66.28% to 89.97% |
| > 97.68 | 37.5 | 15.20% to 64.57% | 82 | 68.56% to 91.42% |
| > 98.11 | 37.5 | 15.20% to 64.57% | 84 | 70.89% to 92.83% |
| > 98.43 | 37.5 | 15.20% to 64.57% | 86 | 73.26% to 94.18% |
| > 99.08 | 37.5 | 15.20% to 64.57% | 88 | 75.69% to 95.47% |
| > 99.87 | 37.5 | 15.20% to 64.57% | 90 | 78.19% to 96.67% |
| > 100.3 | 31.25 | 11.02% to 58.66% | 90 | 78.19% to 96.67% |
| > 100.5 | 31.25 | 11.02% to 58.66% | 92 | 80.77% to 97.78% |
| > 100.7 | 25 | 7.266% to 52.38% | 92 | 80.77% to 97.78% |
| > 100.7 | 25 | 7.266% to 52.38% | 94 | 83.45% to 98.75% |
| > 100.8 | 18.75 | 4.047% to 45.65% | 94 | 83.45% to 98.75% |
| > 101.3 | 12.5 | 1.551% to 38.35% | 94 | 83.45% to 98.75% |
| > 102.1 | 6.25 | 0.1581% to 30.23% | 94 | 83.45% to 98.75% |
| > 102.4 | 0 | 0.000% to 20.59% | 94 | 83.45% to 98.75% |
| > 102.9 | 0 | 0.000% to 20.59% | 96 | 86.29% to 99.51% |
